# A randomised study to assess the nicotine pharmacokinetics of an oral nicotine pouch and two nicotine replacement therapy products

**DOI:** 10.1038/s41598-022-10544-x

**Published:** 2022-04-28

**Authors:** David Azzopardi, James Ebajemito, Michael McEwan, Oscar M. Camacho, Jesse Thissen, George Hardie, Richard Voisine, Gavin Mullard, Zvi Cohen, James Murphy

**Affiliations:** 1 BAT (Investments) Ltd, Research and Development, Regents Park Road, Southampton, SO15 8TL UK; 2 Imperial Tobacco Canada, 3711 Saint-Antoine Street West, Montreal, QC H4C 3P6 Canada; 3 BAT (Holdings) Ltd, Globe House, 4 Temple Place, London, WC2R 2PG UK; 4 R.J. Reynolds Tobacco Company, 950 Reynolds Blvd, Winston-Salem, NC 27105 USA

**Keywords:** Chemical biology, Pharmacokinetics, Pharmacology, Pharmacokinetics

## Abstract

Nicotine replacement therapies (NRTs) are intended for short-term use to help cigarette smokers to quit. Some smokers find NRTs ineffective or seek a more satisfactory source of nicotine. Tobacco-free oral nicotine pouch (NP) products have emerged as a potential reduced risk product compared with cigarettes and other tobacco products. In a randomised crossover clinical study, thirty-four healthy adult smokers were enrolled and their nicotine C_max_ and AUC_0-T_ determined for three 4 mg nicotine products (NP, gum, lozenge) under fasting conditions. The NP, lozenge and gum mean C_max_ values were 8.5, 8.3 and 4.4 ng/mL, AUC_0-T_ values were 30.6, 31.5 and 14.3 ng*h/mL, respectively. The NP showed similar nicotine bioavailability to the lozenge (*p* = 0.6526 (C_max_), *p* = 1.0000 (AUC_0-T_)), and superior bioavailability to the gum (*p*  < 0.0001 for C_max_ and AUC_0-T_). Compared with the lozenge, the NP demonstrated greater product satisfaction with a higher number of positive responses to subjective satisfaction questions. All products were judged to be well-tolerated; the incidence of minor adverse events was lower for the NP (18.2%) than the lozenge (33.3%) or gum (18.8%). In summary, NPs may provide smokers with a more satisfying alternative nicotine source as compared to the reference NRTs.

*Study Registry/Registered Trial No:* ISRCTN/ISRCTN65708311.

## Introduction

The health risks of cigarette smoking, including its strong link to lung and cardiovascular disease, are well known^1^. Chronic smoking-related disorders are a result of the long-term inhalation of tobacco smoke, which contains more than 6500 identified compounds^2^ and at least 150 toxicants^3^. Indeed, it is now commonly accepted that the majority of smoking-related diseases are not due to nicotine^1,4,5^, which, although highly addictive^6^, is relatively harmless at the levels consumed through use of commercial tobacco and nicotine products in the intended manner^7^.

Undoubtedly, the best way to reduce the risk of developing smoking-related chronic disease and increase life expectancy is to stop smoking cigarettes altogether^7–9^; however, quitting smoking is not so easy. One study has estimated that it may take up to 30 attempts before a smoker quits successfully^10^.

Various approaches, including substitute products and clinical support programs, have been developed to help smokers quit. Nicotine replacement therapy (NRT) was reported first in the form of a chewing gum, which was shown to prevent or reduce certain symptoms from smoking abstinence^11^, and soon thereafter as a transdermal nicotine patch^12^. Now regulated as medicinal products, NRTs are intended as a short-term intervention to help an individual switch from cigarette smoking to complete abstinence by replacing the nicotine previously supplied by cigarettes. A recent systematic review of 133 trials on different NRTs (e.g., transdermal patches, chewing gums, nasal sprays, inhalers, lozenges or sublingual tablets) has shown that these products can increase by 50%–60% the rate of successful cessation among smokers who are motivated to quit^13^. Nevertheless, NRTs do not increase the rate of successful cessation for many smokers who try them.

On inhalation of cigarette smoke, nicotine rapidly enters the bloodstream and is transported throughout the body, triggering acetylcholine receptors in the brain involved in mood, concentration and relaxation^6^. The slower and potentially reduced uptake of nicotine from NRTs relative to cigarettes^14–16^ may not satisfy nicotine cravings sufficiently to contribute to successful smoking cessation.

Recognising that some individuals cannot easily stop smoking, the US Institute of Medicine formulated a detailed regulatory proposal for “tobacco harm reduction”—namely, the use of potentially reduced risk tobacco and nicotine products instead of cigarette smoking – 20 years ago^17^. This concept is now supported by respected public health authorities as a way to prevent or reduce the health risks of cigarette smoking for individuals and among populations ^5,7,18^. A number of potentially reduced risk tobacco and nicotine products, such as e-cigarettes and tobacco heating products have been developed^19,20^. In the United States, the Food and Drug Administration (FDA) has defined a modified risk tobacco product (MRTP) as “any tobacco product that is sold or distributed for use to reduce harm or risk of tobacco-related diseases associated with commercially marketed tobacco products”, and published guidance for how a product may achieve MRTP status^21^. Via this pathway, the FDA granted Modified Risk status to eight brands of Swedish snus, a smokeless tobacco product, in 2019^22^.

Prior to achieving MRTP status, Swedish snus had long been recognised to have reduced health risks relative to cigarette smoking based on extensive epidemiological studies^23–29^. Snus is a moist tobacco product containing ground or cut tobacco that comes in either a pouch or loose-leaf format. It is placed under the top lip next to the gum, where the nicotine released from the product is absorbed through the oral mucosa. The lower health risks from snus as compared with cigarettes stem from the absence of tobacco combustion and a lack of direct lung exposure to toxicants during snus use. In particular, Sweden, where 20% of the population uses snus as compared with 5% who smoke, has one of the lowest rates of smoking-related disease in Europe^30^, even though overall tobacco product use is similar to that in other European countries^29^. This is commonly known as the “Swedish Experience”^31^ and has been supported by comprehensive epidemiological data showing that use of snus is not a significant risk factor for developing lung cancer or cardiovascular disease^29^.

Similar in concept to portion snus but without tobacco, ‘modern’ oral nicotine pouches (NPs) have been commercially available since the mid-2010s in many countries. Like snus, these tobacco-free NPs, which contain pharmaceutical-grade nicotine, are placed between the upper lip and the gum, where they release nicotine and flavourings; the nicotine is then absorbed through the oral mucosa. A recent study has shown that NPs have potential as reduced risk nicotine products with significantly fewer toxicants^32^ and reduced toxicological effects^33^ as compared with cigarette smoke and snus.

For nicotine products to be satisfactory nicotine sources for cigarette smokers seeking an alternative to conventional cigarettes, they must not only provide nicotine in sufficient and timely quantities, but also provide other qualities enjoyed by smokers, such as sensorial satisfaction. Therefore, we have conducted a randomised crossover study to compare, for the first time to our knowledge, nicotine pharmacokinetics and product satisfaction between an NP and two established NRTs (nicotine gum and lozenge). The primary aim was to compare nicotine bioavailability between the NP and each reference NRT under fasting conditions specified by the Health Canada Therapeutic Products Directorate (TPD)^34^. Product satisfaction, likability, safety and tolerability of the products were also assessed. We discuss the implications of our findings on the potential of oral NPs to act as a satisfactory nicotine source for smokers seeking alternatives to conventional cigarettes.

## Materials and methods

### Study design

This was a single-center, randomised, three-product, three-period, six-sequence, crossover, single-dose study, in which healthy adult smokers received one of three investigational products (oral NP, nicotine gum, or nicotine mini lozenge) during each study period. Participants fasted overnight for a minimum of 8 h before each study period, when they received one study product and underwent 12-h blood sample collection for PK analysis of nicotine.

The study was carried out at a single clinical site in Montreal, Quebec, Canada. Ethical and competent authority clinical trial approvals were given by a local Institutional Review Board (Advarra, Ontario, Canada) and by Health Canada (NOA 249515) before study commencement respectively. The study was conducted in compliance with the Declaration of Helsinki, the ICH Guideline E6 for GCP, the FDA GCP Code of Federal Regulations Title 21 (part 56), European Union regulation EU 536/2014, and the Tri-Council Policy Statement (Canada). Written informed consent was obtained from all participants before their enrolment and before undergoing any study procedures, including screening assessments. Participants were free to withdraw from the study at any time without prejudice.

### Study participants

The study aimed to recruit 36 participants. Potential participants attended a screening session to assess eligibility, which was reconfirmed upon admission to the clinical site for the randomised study. The main inclusion criteria were informed consent; in good health; aged 19 years to 55 years inclusive; body mass index of 18.5–30.0 kg/m^2^ inclusive; minimal body weight of 50 kg; not pregnant or likely to become pregnant if female; primary tobacco product use of combustible or roll-your-own cigarettes; a smoker of 10 cigarettes (> 6 mg ISO tar) per day for at least 6 months; willingness to abstain from nicotine and tobacco products (except for the study product provided) from 24 h before the first study period until the end of the study; positive urine cotinine test (≥ 200 ng/mL) at screening and before the first study period, and successful completion of the product use training session prior to the first study period.

The main exclusion criteria were pregnancy or breast-feeding; presence or history of significant disease or surgery that might affect drug bioavailability; history of significant cardiovascular, pulmonary, haematologic, neurological, psychiatric, endocrine, immunologic, or dermatologic disease; presence of ECG abnormalities at screening; maintenance therapy with any drug (except for hormonal contraceptives or hormone replacement therapy) or significant history of drug dependency or alcohol abuse (> 3 units of alcohol per day, intake of excessive alcohol, acute or chronic); clinically significant illness in the 28 days before the first study period; use of prescription drugs (except for hormonal contraceptives or hormone replacement therapy) in the 28 days before the first study period; use of any medication or substance that aids in smoking cessation; history of tuberculosis; positive screening results to HIV, hepatitis B surface antigen or hepatitis C virus tests; postponement of a decision to quit using tobacco- or nicotine-containing products in order to participate in this study, and an attempt to quit using tobacco- or nicotine-containing products in the 28 days before the first study period. For full details of inclusion and exclusion criteria, refer to the Supplementary Information or https://www.isrctn.com/ISRCTN65708311.

### Investigational products

The Test product was an NP (Peppermint NP, 4 mg nicotine; BAT); the reference NRT products were a nicotine gum (Reference-1; nicotine polacrilex, 4 mg; Nicorette®) and a nicotine mini-lozenge (Reference-2; nicotine polacrilex, 4 mg; Nicorette®). Both reference products were commercially available at the time of the study.

### Randomisation and blinding

Participants (n = 36) were randomised to one of six sequences of the three study products (each product use occurring in a study period), using a computer program. The random allocation of each sequence of product administration to each subject was performed so that the study was balanced. The randomisation code was not revealed until the bioanalytical tables were finalised. The statistician and pharmacokineticist remained blind to the product used on each study day until final analyses had been performed.

### Study procedures

**Figure 1:**
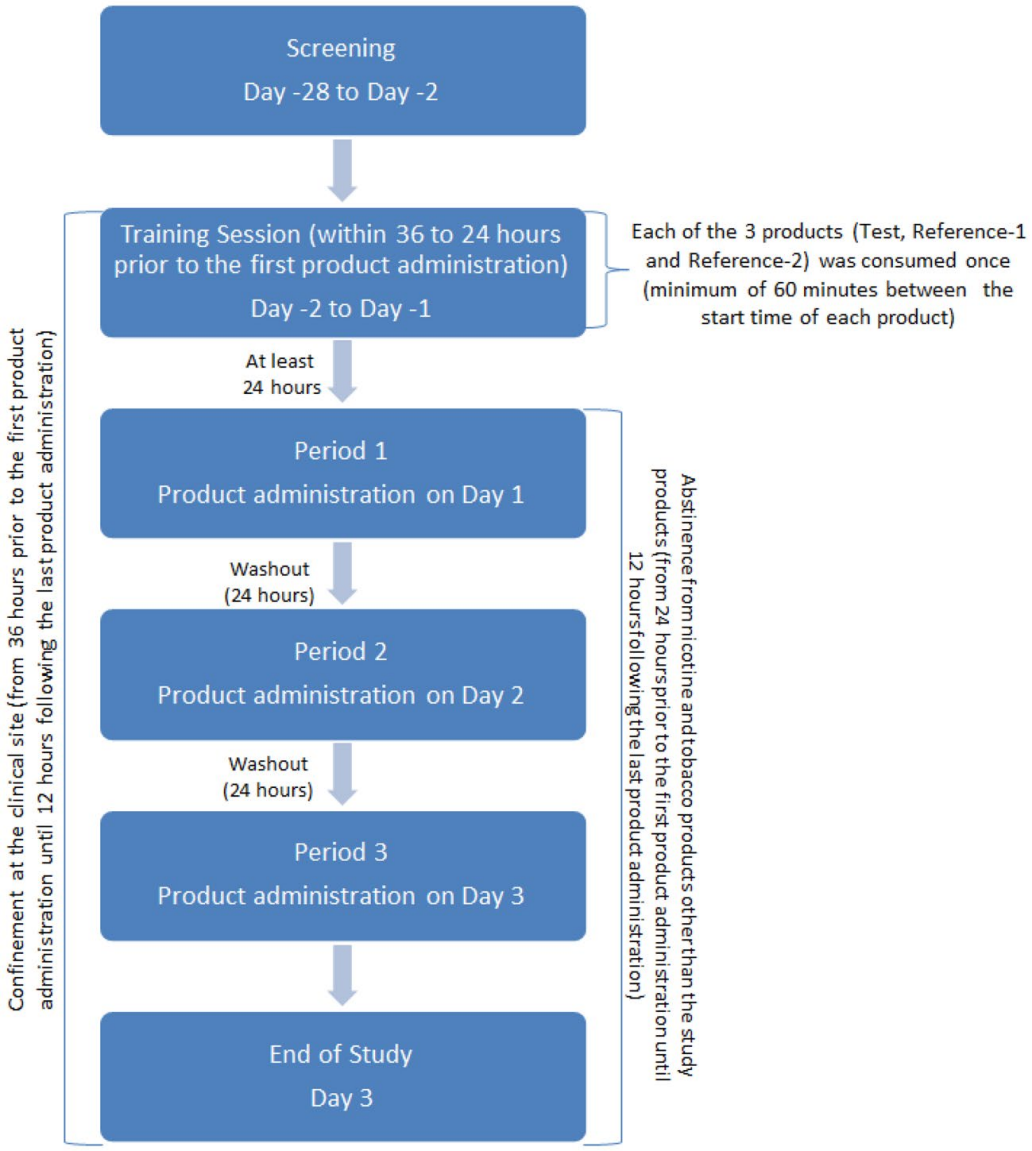
Study scheme.

Eligible participants enrolled at screening were admitted to the clinical site within 28 days of screening and at least 36 h (Day –2) before the first study period. They were confined to the study site until the last assessments after the third study period on Day 3 (Fig. 1). On admission, eligibility criteria were reconfirmed, and participants underwent vital signs assessments, a physical examination if deemed necessary, and an oral mucosa examination. They completed a training session 24–36 h before the first period, during which they were allowed to try each of the three products once in accordance with the respective manufacturer’s instructions. A minimum of 60 min separated the trial use of each product. Only participants who successfully completed the training session could continue in the study, i.e., participants were able to follow the usage instructions and did not experience significant AEs. From 24 h before the first study period until the end of the third study period, participants abstained from using any nicotine or tobacco products other than the product in each study period. Other restrictions prior to and during the study are given in Supplementary Information.

#### Daily product administration schedule

Food and intake of fluid other than water were controlled during the confinement period for all participants. Products were administered in the morning; therefore, participants fasted overnight (no food or drink except water) for a minimum of 8 h before administration of each study product (excluding the training session). Fasting continued for at least 4 h after product administration, following which a standardised lunch was served. A supper and a light snack were served at appropriate times thereafter, but not until at least 9 h after product administration. Water was provided as needed until 1 h before product administration, and allowed from 1 h after administration. No water was administered with the study product. Participants remained seated or maintained minimal ambulatory movement for the first 4 h after product administration, avoiding both vigorous exertion and complete rest.

In each study period, participants used a single product according to the randomisation schedule. Before administration, the participants were re-instructed on how to use the assigned product.

#### Administration of test product

The oral NP was placed in the participant’s mouth by study staff. Participants were instructed to position the pouch between their top lip and their gum for 60 min. Subjects were asked to swallow their saliva as needed during this period. The pouch was not to be swallowed whole, chewed or broken. At the end of the 60-min period, the study staff removed the pouch from the subjects’ mouth. The used pouch was retained for analysis of residual nicotine content.

#### Administration of reference-1

Participants took the nicotine gum directly from the dosing container by holding the container to their mouth; they did not touch it with their hands. They were instructed to bite the gum and position it between their gum and cheek for 1 min. They continued to repeat the previous step (bite and park) for 30 min, swallowing their saliva as needed. At the end of the 30-min period, they removed the gum from their mouth. The gum was not to be swallowed whole. The spent gum was retained for analysis of residual nicotine content.

#### Administration of reference-2

Participants took the nicotine lozenge directly from the dosing container by holding the container to their mouth; they did not touch it with their hands. They were instructed to occasionally move the lozenge from one side of the mouth to the other until it dissolved completely (~ 10 min). Participants were asked to minimise swallowing and to not spit out their saliva during this period. They notified the study staff as soon as the lozenge was completely dissolved and the study staff performed a mouth check to ensure that the product was consumed. The lozenge was not to be swallowed whole, chewed or broken.

### Blood sampling for nicotine PK assessment

Venous blood samples were collected from an intravenous cannula inserted into a forearm vein at the start of each study day or, if necessary, by direct venipuncture. Samples for PK measurements were collected before and at 5, 10, 20, 30, 40, 50 min, and at 1, 1.25, 1.5, 2, 3, 4, 5, 6, 7, 8, 9, 10, 11, and 12 h relative to the start of product administration.

Blood samples were collected in K_2_EDTA vacutainers and centrifuged as soon as possible (within 60 min) at approximately 1500* g* at 4 °C for 10 min. The plasma was separated into two aliquots and stored at –20 °C until shipment to the bioanalytical laboratory. The time from blood sample collection to plasma aliquot storage did not exceed 90 min.

Plasma nicotine analysis was performed by Altasciences (Laval, Quebec, Canada). Plasma samples were assayed for nicotine using liquid chromatography with tandem mass spectrometry detection (LC–MS/MS). Sample pre-treatment involved the protein precipitation extraction of nicotine from 0.150 mL of human plasma; nicotine-D4 was used as the internal standard. The compounds were identified and quantified using reversed-phase LC–MS/MS detection over a theoretical concentration range of 0.200 ng/mL to 100.000 ng/mL. The concentrations were calculated using peak ratios and the linearity of the calibration curve was determined using a weighted (1/x^2^) linear (y = mx + b) least squares regression analysis for nicotine.

### Subjective effects assessments

At the end of the product usage period, participants completed a product appreciation questionnaire in order to evaluate participant subjective effects of product use. Questions included ‘Was the product satisfying?’, ‘Did it taste good?’ and ‘Did you enjoy the sensations in the mouth?’, and were answered using a 7-point scale, whereby 1 = ‘Not at all’ and 7 = ‘Extremely’. In addition, subjects were asked ‘How much did you like the product?’, which was scored using a scale from 0 to 100, whereby 0 = ‘Not at all’ and 100 = ‘Extremely’.

### Residual nicotine assessment

Used pouches and gums were retained for nicotine analysis to enable calculation of transferred nicotine. Testing was performed by Labstat International Inc. (Kitchener, Canada) on a “per unit” basis using an entire pouch (~ 0.7 g) or gum (~ 1.25 g) per replicate testing. The total weight of the gum and pouch material/contents were recorded. Pouch test items were cut in half and the pouch material and pouch contents were added to an extraction vessel for analysis. The pouch material and contents were spiked with nicotine-D3 internal standard and extracted with 100 mM ammonium acetate solution using an ultrasonic bath. The extract was centrifuged and an aliquot of the supernatant analysed by LC–MS/MS.

The gum was cut into four roughly equal pieces and these were shaken with 40 mL of hexane, 1 mL of internal standard and 39 mL of extraction solvent for 30 min. The two phases separate after extraction and an aliquot of the aqueous layer (water/acetonitrile/acetate buffer extract) was spiked with internal standard, extracted, centrifuged and analysed as per the pouch samples.

### Safety assessments

Safety assessments included symptom-oriented physical examination, oral mucosa examination, vital signs, clinical laboratory tests, and adverse event (AE) monitoring. The monitoring period for AEs extended from the pretrial evaluation until the collection of the last blood sample of the study. Full details of safety assessment and definitions of AEs are given in the Supplementary Information.

At the end of the 12-h PK sample collection on study Day 3, participants underwent clinical laboratory testing, oral mucosa examination and a physical examination if deemed necessary. After medical approval, they were discharged from the clinic.

### Study outcomes

The primary outcome was the comparative bioavailability of nicotine from the Test and Reference products, as assessed by the maximum observed concentration of nicotine (C_max_) occurring at time T_max_, and the area under the plasma concentration–time curve calculated from 0 min to the time of the last measurement (AUC_0-T_). Secondary outcomes were the safety and tolerability of the Test and the two Reference products among healthy smokers.

### Sample size

For a Williams crossover design with three products and six sequences, and an overall standard deviation (SD) of paired differences of 0.129 (comparable to an intra-participant coefficient of variation [CV] of 13%), it was calculated that a minimum of 30 participants would be needed to detect an 11% (0.11) difference between mean values with a statistical power of at least 80% and a significance level of 5%. To allow for a potential drop-out rate of 20%, the study aimed to recruit 36 participants.

### Analysis populations

The safety population included all subjects who received at least one of the products. Subjects who received at least one of the investigational products were included in the PK analysis. Subjects who did not complete the sampling schedule of one or more study periods were included in the PK analysis for only the PK parameters that were judged not to be affected by the missing sample(s).

If a pre-dose concentration was detected, the subject’s data was included in the PK population if the pre-dose concentration was equal to or less than 5% of the C_max_ value of the corresponding period. If the pre-dose concentration was greater than 5% of the C_max_ value, the subject was excluded from the PK population for the corresponding period. In the case where more than 10% of subject observations for the whole study population exhibit positive pre-dose concentrations higher than 5% of their respective C_max_ (irrespective of the product received), observed nicotine concentrations were adjusted for baseline nicotine (“baseline-adjusted”) for all subjects including those subjects whose positive pre-dose concentrations are lower than 5% of their respective C_max_ (irrespective of the product received).

### Data analyses

Descriptive statistics were calculated for nicotine concentrations at each individual time point and for all PK parameters. Individual concentrations, actual sampling times, and PK parameters were summarised per product using the following descriptive statistics: number of observations (N), minimum, arithmetic mean, geometric mean, median, maximum, SD and CV. Individual nicotine concentration data and derived concentrations were calculated using software (Phoenix® WinNonlin® version 8.0).

We compared differences in nicotine bioavailability between the Test product and each Reference product as follows: (1) the *P*-value for the 2-sided test of Test versus Reference paired difference for the log-transformed parameter C_max_ was assessed against a significance level of 0.0125; and (2) the *P*-value for the 2-sided test of paired Test versus Reference difference for the log-transformed parameter AUC_0-T_ was assessed against a significance level of 0.0125.

Bioavailability between the Test product and Reference product was compared using a mixed-effect analysis of variance (ANOVA) model with products, periods, sequences as fixed effects and participants nested within sequences as random effects. Product comparison was conducted by using the fitted model with overall significance level of 0.05, which after Bonferroni adjustment of multiple comparisons results in a significance level of 0.0125 per comparison.

We also conducted a secondary analysis in which statistical inference of nicotine was based on a bioequivalence approach using the following standards: (1) the ratio of geometric least-square means (LSmeans) calculated from the exponential of the difference between the Test and each Reference product for the log-transformed parameter C_max_ must be within the 80%–125% bioequivalence range; (2) the ratio of geometric LSmeans with corresponding 90% CI calculated from the exponential of the difference between the Test and each Reference product for the log-transformed parameter AUC_0-T_ must be within the 80%–125% bioequivalence range.

## Results

### Study participants

Recruitment of potential participants for the study commenced on 20th July 2020. Screening commenced on 27th July 2020, followed by the clinical phase from 11th August to 24th September 2020. The study planned to include 36 participants, but owing to recruitment challenges only 34 of the 132 subjects screened were enrolled and randomised, having passed the screening procedures and successfully completed the training session. Of these 32 (94.1%) participants completed the study as per the protocol, while 2 (5.9%) withdrew on Day 1 and discontinued the study. All 34 enrolled participants received at least one of the products and comprised the safety population. The PK population comprised the 32 participants who completed the whole study.

The majority of participants in the safety population were male (91.2%), white (94.1%), and not Hispanic or Latino (94.1%). The age of the participants ranged from 20 to 54 years (mean ± SD, 33.9 ± 9.12 years) and body mass index ranged from 18.9 to 29.9 kg/m^2^ (24.86 ± 3.145 kg/m^2^). Participant demographics and baseline characteristics were comparable among randomisation sequences. The demographic characteristics of the safety population are summarised in Table 1.

**Table 1:** Demographic and baseline characteristics of the safety population.

Characteristic	Overall (N = 34)
**Age, years**	
Mean (SD)	33.9 (9.12)
Median	31.5
Min, Max	20, 54
**Sex, n (%)**	
Male	31 (91.2)
Female	3 (8.8)
**Ethnicity, n (%)**	
Hispanic or Latino	2 (5.9)
Not Hispanic or Latino	32 (94.1)
**Race, n (%)**	
Black or African American	1 (2.9)
White	32 (94.1)
Other	1 (2.9)
**Weight, kg**	
Mean (SD)	75.87 (12.764)
Median	76.45
Min, Max	51.5, 99.1
**Height, cm**	
Mean (SD)	174.31 (7.856)
Median	174.25
Min, Max	159.8, 189.4
**Body mass index, kg/m**^**2**^	
Mean (SD)	24.86 (3.145)
Median	25.00
Min, Max	18.9, 29.9

*Max* maximum, *Min* minimum, *SD* standard deviation.

### Nicotine pharmacokinetics

Pre-dose nicotine concentrations were above the LLOQ (0.200 ng/mL) in 51% (49/96) of participant observations and higher than 5% of the respective C_max_ in 26% (25/96) of participant observations. Nicotine PK parameters were therefore calculated using both baseline-adjusted and unadjusted nicotine concentrations. There was no difference in the statistical analysis between the two data sets (see below); therefore, only the unadjusted parameters are presented herein.

**Figure 2:**
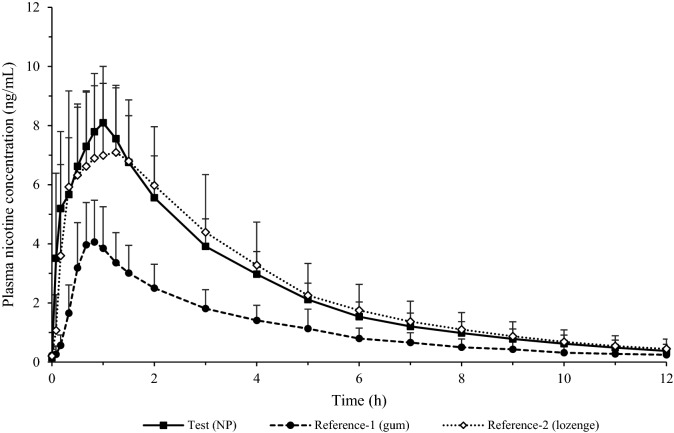
Baseline-unadjusted mean plasma nicotine concentrations among adult smokers (N = 32) after single oral product use of three nicotine products under fasting conditions.

The mean ± SD plasma nicotine concentration–time profiles following single oral product use of various nicotine products under fasting conditions in adult smokers are displayed in Fig. 2. Overall, the three products showed a similar nicotine concentration profile with plasma nicotine levels peaking within the first hour after product administration; however, the peak of nicotine concentration reached was much lower for the nicotine gum than for the oral NP or nicotine lozenge.

**Figure 3:**
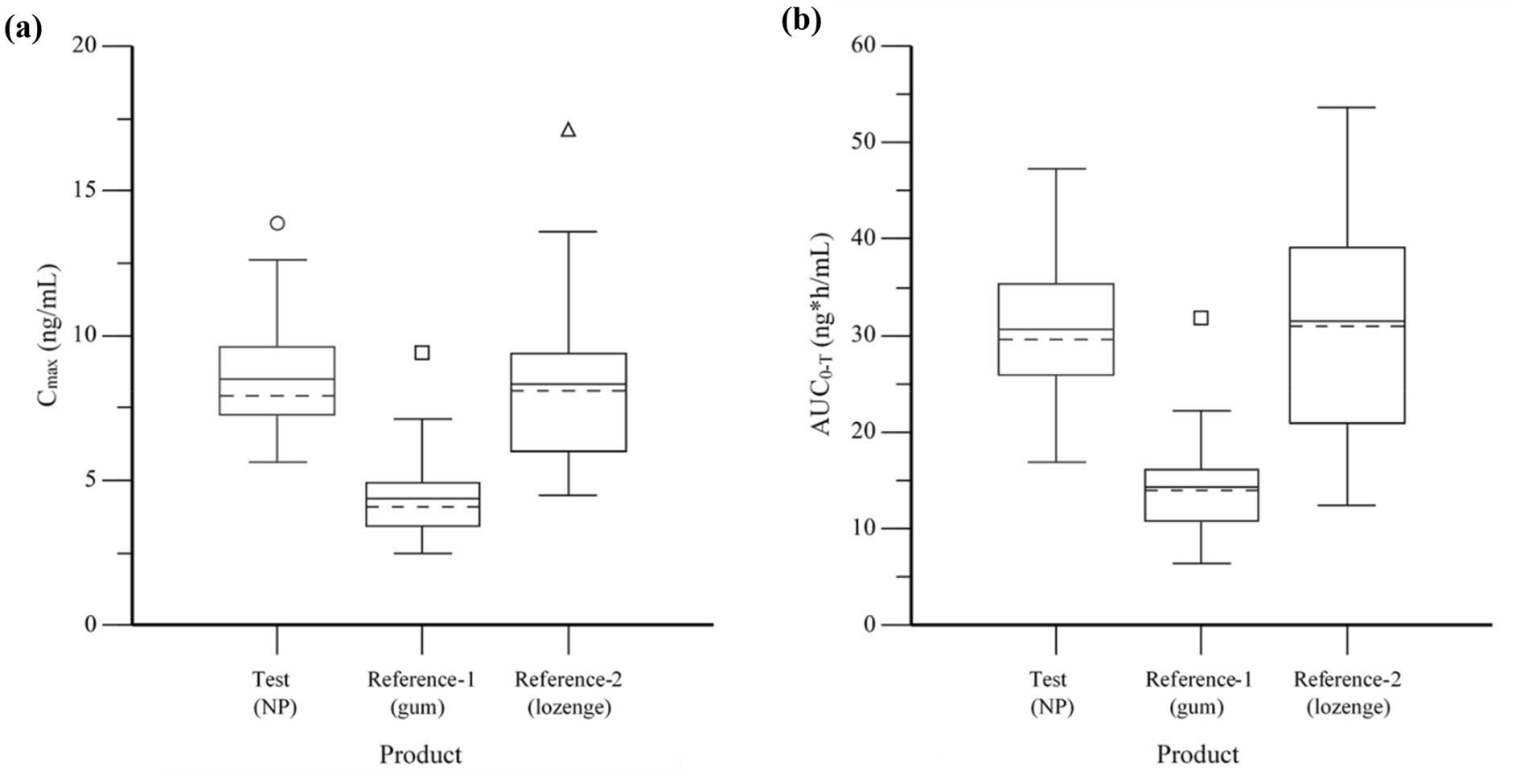
Box-and-whiskers plot of plasma nicotine C_max_ (**a**) and AUC_0-T_ (**b**) among adult smokers after single oral product use of three nicotine products under fasting condition. Arithmetic mean values are shown as solid lines and median values as dashed lines.

Nicotine PK parameters are summarised in Table 2, and box-and-whiskers plots of the key parameters C_max_ and AUC_0-T_ are presented in Fig. 3. Time to maximum concentration was comparable between the Test product and both Reference products with a median (min–max) T_max_ value of 60 min (5–90 min) for the oral NP, 50 min (30–75 min) for nicotine gum, and 60 min (10–180 min) for nicotine lozenge.

**Table 2:** Plasma nicotine PK parameters measured among adult smokers after single oral product use of three nicotine products under fasting conditions.

Parameter	Statistic	Test (NP, N = 32)^a^	Reference-1 (gum, N = 32)^a^	Reference-2 (lozenge, N = 32)^b^
C_max_ (ng/mL)	Mean (SD)	8.5 (2.06)	4.4 (1.48)	8.3 (3.00)
	GM (CV %)	8.291 (23.4)	4.150 (31.2)	7.821 (36.0)
AUC_0-T_ (ng*h/mL)	Mean (SD)	30.6 (7.33)	14.3 (5.02)	31.5 (11.48)
	GM (CV %)	29.744 (24.5)	13.526 (34.8)	29.434 (40.3)
T_max_ (h)	Median (Min–Max)	1.00 (0.08–1.50)	0.83 (0.50–1.25)	1.00 (0.17–3.00)
T_1/2_ (h)	Mean (SD)	2.7 (0.77)	3.0 (0.88)	2.7 (0.57)

*CV* coefficient of variation, *GM* geometric mean, *Max* maximum, *Min* minimum, *SD* standard deviation.

^a^N = 31 for T_1/2_ (the terminal part of the log concentration–time curve could not be adequately estimated for 1 participant).

^b^N = 29 for T_1/2_; (the terminal portion of the log concentration–time curve could not be adequately estimated for 3 participants).

Mean C_max_ values were not similar between the oral NP and nicotine gum at 8.5 and 4.4 ng/mL, respectively; similarly, mean AUC_0-T_ also differed between these two products at 30.6 and 14.3 ng*h/mL, respectively. By contrast, mean C_max_ and AUC_0-T_ were similar between the oral NP and nicotine lozenge (8.5 vs 8.3 ng/mL, and 30.6 vs 31.5 ng*h/mL, respectively).

Nicotine plasma half-life was found to be similar among the three products with a mean T_1/2_, respectively, of 2.7, 3.0 and 2.7 h for the NP, gum and lozenge, respectively.

We used primary and secondary statistical analysis to assess comparative bioavailability among the products (see “Materials and methods”). In both unadjusted and baseline-nicotine-adjusted analyses, C_max_ and AUC_0-T_ were significantly different between the oral NP and nicotine gum (both *p*  < 0.0001) (Table 3). In addition, the NP/gum ratios of geometric LSmeans of C_max_ and AUC_0-T_ were outside the predefined bioequivalence acceptance range of 80%–125%.

**Table 3:** Statistical analysis of plasma nicotine PK parameters among adult smokers after single oral product use of three nicotine products under fasting condition.

Parameters	Comparison	IPCV (%)	Geometric mean ratio (90% CI)	*p *value
C_max_, ng/mL	Test (NP) vs Gum	20.4	200.7 (184.5–218.5)	< 0.0001
	Test (NP) vs Lozenge	20.4	106.5 (97.9–115.9)	0.6526
AUC_0-T_, ng*h/mL	Test (NP) Gum	17.1	220.7 (205.5–236.9)	< 0.0001
	Test (NP) vs Lozenge	17.1	101.4 (94.4–108.9)	1.0000

*CI* confidence interval, *IPCV* intra-participant coefficient of variation.

By contrast, C_max_ (*p* = 0.6526) and AUC_0-T_ (*p* = 1.0000) were not significantly different between the oral nicotine pouch and nicotine lozenge. Likewise, the NP/lozenge ratios of geometric LSmeans of C_max_ and AUC_0-T_ were between 80 and 125%, and the 90% confidence intervals of AUC_0-T_ were contained within the predefined bioequivalence acceptance range of 80%–125%. The intra-participant variability associated with unadjusted plasma nicotine levels was 20.4% for C_max_, and 17.1% for AUC_0-T_ (Table 3).

### Residual content analysis

Notably, the residual nicotine content was highly variable among participants, ranging from 0.40 to 2.65 mg in used pouches and 1.04 to 2.84 mg in the used gums. However, the mean residual content was approximately 2.0-fold higher for the gum than for the pouch (2.46 and 1.26 mg, respectively), suggesting a substantial difference in nicotine exposure from the two products. Using the measured nicotine content of the unused pouch (3.35 mg) and gum (3.69 mg), the corresponding mean percentage extraction of nicotine was 62% and 33% respectively.

### Subjective effects assessment

Subjective effects responses are summarised in Table 4. For the question ‘How much did you like the product?’, the NP had the highest number of positive (> 55 score) and lowest number of negative (< 45) responses with 18 (54.5%) and 10 (30.3%) respectively, compared with 3 (9.4%) and 24 (75.0%) respectively for the gum, and 6 (18.8%) and 24 (75.0%) respectively for the lozenge. For the questions ‘Was it satisfying?’, ‘Did it taste good?’ and ‘Did you enjoy the sensations in the mouth?’, the gum had the highest number of positive responses (5–7 scores) with 16 (50.0%), 22 (68.8%) and 19 (59.4%) respectively, followed by the NP with 13 (39.4%), 12 (36.4%) and 9 (27.3%) respectively, and finally the lozenge with 5 (15.6%), 3 (9.4%) and 2 (6.3%). For these three questions, the lozenge had the highest number of negative responses (1–3 scores) with 21 (65.6%), 24 (75.0%) and 25 (78.1%) respectively, followed by the NP with 9 (27.3%), 10 (30.3%) and 15 (45.5%) respectively, and finally the gum with 3 (9.4%), 2 (6.3%) and 4 (12.5%).

**Table 4:** Summary of subjective effects assessment.

Question	Test (NP)	Reference-1 (gum)	Reference-2 (lozenge)
**How much did you like the product?**^a^			
< 45	10 (30.3)	24 (75.0)	24 (75.0)
45–55	5 (15.2)	5 (15.6)	2 (6.3)
> 55	18 (54.5)	3 (9.4)	6 (18.8)
**Was it satisfying?**^b^			
1–3	9 (27.3)	3 (9.4)	21 (65.6)
4	11 (33.3)	13 (40.6)	6 (18.8)
5–7	13 (39.4)	16 (50.0)	5 (15.6)
**Did it taste good?**^b^			
1–3	10 (30.3)	2 (6.3)	24 (75.0)
4	11 (33.3)	8 (25.0)	5 (15.6)
5–7	12 (36.4)	22 (68.8)	3 (9.4)
**Did you enjoy the sensations in the mouth?**^b^			
1–3	15 (45.5)	4 (12.5)	25 (78.1)
4	9 (27.3)	9 (28.1)	5 (15.6)
5–7	9 (27.3)	19 (59.4)	2 (6.3)

Data are presented as n (%). Number of subjects (denominator) = 33 for NP and 32 for gum and lozenge.

^a^Original scale was from 0 (not at all) to 100 (extremely).

^b^Original scale was from 1 (not at all) to 7 (Extremely).

### Safety evaluation

During the study, 33 (97.1%) participants used the oral NP, 32 (94.1%) used the nicotine gum, and 33 (97.1%) participants used the nicotine lozenge product. Overall, 40 AEs were reported, 29 of which were product use emergent AEs (PEAEs). Among the safety population, 18 (52.9%) participants experienced at least 1 PEAE during the study, and 14 (41.2%) participants experienced at least 1 PEAE that was considered related to any study product.

Of the 29 PEAEs, 7 occurred after administration of the NP, 9 after administration of the nicotine gum, and 13 after administration of the nicotine lozenge. Most of the PEAEs were considered to be product-related (23/29; 79.3%). The incidence of PEAEs and product-related PEAEs was higher for the lozenge (33.3% and 30.3%, respectively) than for the pouch (18.2% and 12.1%, respectively) or gum (18.8% and 12.5%, respectively). All PEAEs experienced during the study were resolved by the end of the study. The majority of PEAEs were mild in intensity (28/29; 96.6%). Dizziness was the most common PEAE, being reported by 2 (6.1%) participants after receiving the nicotine pouch, 1 (3.1%) participant after receiving nicotine gum, and 3 (9.1%) participants after receiving the nicotine lozenge. Nausea was reported by 3 (9.1%) participants after receiving the nicotine lozenge. Throat irritation was reported by 1 (3.1%) participant after receiving the nicotine gum and 2 (6.1%) participants after receiving the nicotine lozenge.

The one recorded PEAE of severe intensity was fainting due to catheter reinsertion, which occurred in period 1 for a participant receiving the nicotine lozenge. The PEAE was considered unrelated to product administration and was resolved within a minute of onset. No serious AEs occurred in the study. Moreover, no participant was withdrawn by the investigator due to a PEAE (safety reasons).

### Discussion

The present study has evaluated nicotine pharmacokinetics for a ‘modern’ oral NP in comparison to two established NRTs, nicotine gum and nicotine lozenge. Based on guidance from the Health Canada TPD, the study was carried out in the fasted state^34^. The study found that the NP delivers nicotine effectively. The mean C_max_ and AUC_0-T_ values for the pouch were 8.5 ng/mL and 30.6, ng*h/mL, respectively; these values were similar to those of the lozenge (8.3 ng/mL (*p* = 0.6526) and 31.5 ng*h/mL (*p* = 1.0000) respectively), but much higher than those of the gum (4.4 ng/mL and 14.3 ng*h/mL, respectively; *p* < 0.0001). Thus, the oral NP had bioequivalence to the nicotine lozenge and was significantly more efficacious than the nicotine gum at delivering nicotine; as a result, the 4 mg NP may provide a satisfactory nicotine source for smokers who quit smoking but want to continue enjoying nicotine through use of a potentially reduced risk product.

The C_max_ and AUC values observed from use of the nicotine gum are lower than several reported values from previous PK studies with nicotine gum^35–38^. Furthermore, studies including a nicotine gum and lozenge (4 mg) have shown similar C_max_ and AUC values between these products^35,36,39^, with values for the lozenge similar to the present findings. However, these studies adopted the use of a metronome to standardise chewing of the gum, typically every 2 s for thirty minutes. In contrast, studies employing the ‘chew and park’ method (as per the Nicorette gum use instructions), had C_max_ values consistent with the results presented from this study^40–42^. The contrasting values from the two different use methodologies suggests that a user of nicotine gum could modulate their nicotine exposure depending on their chewing behaviour.

Cigarette nicotine PK was not assessed within this study; this has been widely reported with C_max_ and AUC typically ranging from ~ 12 to 20 ng/mL and ~ 23 to 31 ng*h/mL (some values converted from ng/mL*min) respectively^40,43–46^. Although the pouch AUC of 30.6 ng*h/mL reported in this study compares favourably to that of cigarettes, the C_max_ of 8.5 ng/mL is approximately half of that resulting from cigarette use. However, NPs come in different strengths, typically ranging from 3 to 11 mg nicotine/pouch, and thus might suit the broad range of individual smokers’ preferred levels of nicotine consumption. Indeed, McEwan et al. demonstrated that NP products with nicotine contents ranging from 6 to 10 mg/pouch used by dual users of cigarettes and snus for 60 min resulted in either greater or similar C_max_ and AUC_0-6 h_ values to that of a cigarette^46^.

Analysis of the spent pouches showed that extraction of nicotine varied substantially among participants. The mean percentage extraction of nicotine was 62%, which is similar to previously levels recently reported for 3 mg (56%) and 6 mg (59%) pouches^47^. By contrast, the gum showed only 33% extraction of nicotine. It has been previously documented that there is residual nicotine left in gum after use^16^, but the present mean value is lower than previous reports of 63%^38^ and 64%^37^ extraction. As discussed earlier, the disparity in nicotine extraction values is likely to be a result of these published studies using the ‘metronome’ method to standardise chewing, whereas subjects in this study used the ‘chew and park’ method, in line with the manufacturer’s instructions. Due to the nature of lozenge use, 100% of nicotine is extracted, but inevitably some of the extracted nicotine will be ingested and subject to first-pass metabolism by the liver^16^. Although more nicotine is extracted from the lozenge than the NP (100% vs 62%), the similar C_max_ and AUC_0-T_ values observed for the NP and lozenge suggests that absorption of released nicotine is greater from the pouch than the lozenge.

Few studies have previously assessed the nicotine pharmacokinetics of oral NPs. In addition to the McEwan et al. study described previously^46^, Rensch et al. compared nicotine PK of various 4 mg NPs with cigarettes in adult smokers^48^. The NP C_max_ values are comparable to the NP C_max_ in this study with geometric means ranging from 9–11 ng/mL, however, AUC_0-T_ values were lower (14–18 ng*h/mL, converted from ng/mL*min), presumably as these were calculated from a shorter 180-min PK session. In a study among snus users, Lunell et al. recently compared the pharmacokinetics of two oral NPs versus a Swedish snus with MRTP status (General, 8 mg nicotine)^47^. They found that AUC_inf_ was 27% smaller and 34% larger, respectively, for 3 mg and 6 mg pouches as compared with snus, and concluded that the higher-dose NP delivers nicotine as quickly and to a similar extent as Swedish snus.

Results of the subjective effects assessment indicate that NPs may appeal to some smokers, scoring higher than both the gum and lozenge for the question ‘How much did you like the product?’, with 55% of subjects giving positive scores. Although the gum scored poorly for this question, it received the highest number of positive responses for the remaining three questions: ‘Was it satisfying?’, ‘Did it taste good?’ and ‘Did you enjoy the sensations in the mouth?’. Given that the gum had a relatively low C_max_ and AUC compared with the NP, lozenge and published cigarette PK data, these results suggest that factors other than nicotine PK play an important role in overall likeability of a nicotine product. In addition, for the ‘‘Did you enjoy the sensations in the mouth?’ question, the familiarity of the gum format and usage may have contributed to it receiving a higher number of positive scores compared with the NP, suggesting that smokers who have not used pouch products before may require some time to become accustomed to the new format and method of product use. Although the NP and lozenge had similar C_max_ and AUC_0-T_ values, the pouch outperformed the lozenge in all four subjective questions, furthermore, suggesting that factors other than nicotine PK are important in the overall likeability of a nicotine product to smokers.

With respect to safety, NPs were generally very well tolerated with no serious AEs noted after administration, while six participants reported minor AEs (dizziness, headache, hot flush, hyperhidrosis and back pain) that resolved quickly. The pouch also seemed to be better tolerated than the lozenge, which had an incidence of product-related AEs of 30.3% as compared with 12.1% for the oral NP.

Our study has several strengths. First, it was conducted under fasting conditions because eating is known to decrease the absorption of nicotine^16^. Second, it was conducted among smokers, allowing us to assess whether NPs can provide nicotine at an acceptable rate and level for this group of tobacco users. Last, the study compared NPs with commercially available NRTs that are approved and accepted smoking cessation aids. The bioequivalence observed between the pouch and the lozenge indicate that nicotine pouches may provide nicotine in sufficient quantity to satisfy many smokers seeking a complete alternative to cigarettes while providing a more likeable product than the reference NRTs. The study also has limitations. Nicotine pharmacokinetics were measured after a single dose of each study product and it is not known whether nicotine delivery will change with continued use of the nicotine products.

In summary, an oral NP was found to provide nicotine bioavailability comparable to that of a commercial nicotine lozenge under fasting conditions, but was better tolerated and received a greater number of positive scores to subjective questions than the lozenge. Taken together with data from a recent survey-based study indicating that some smokers find NPs appealing and may percieve them as a reduced-risk product^49^, and positive subjective responses from smokers during an NP nicotine PK study^48^, these products may act as a nicotine source that provides smokers with a satisfactory and complete alternative to conventional cigarettes.

## Supplementary Information

Supplementary Information.

## Data Availability

All data and materials are available upon reasonable request.
