# Supplementary material for: A randomised study to assess the nicotine pharmacokinetics of an oral nicotine pouch and two nicotine replacement therapy products
Source: Sci Rep. 2022 Apr 28;12:6949. doi: 10.1038/s41598-022-10544-x (PMC9050656; doi:10.1038/s41598-022-10544-x)
Supplement: Supplementary file 1 — Supplementary Information. [file 41598_2022_10544_MOESM1_ESM.docx]

**A randomised study to assess the nicotine pharmacokinetics of an oral nicotine pouch and two nicotine replacement therapy products**

David Azzopardi^1,*^, James Ebajemito^1^, Michael McEwan^1^, Oscar M Camacho^1^, Jesse Thissen^1^, George Hardie^1^, Richard Voisine^2^ , Gavin Mullard^3^, Zvi Cohen^2^ and James Murphy^4^

**Supplementary Information**

# **Inclusion Criteria**

1. Provision of signed and dated informed consent form
2. Stated willingness to comply with all study procedures and availability for the duration of the study
3. Healthy adult male or female
4. If female, meets one of the following criteria:
5. Is of childbearing potential and agrees to use two of the accepted contraceptive regimens from at least 28 days prior to the first study product administration through to at least 30 days after the last dose of study product. An acceptable method of contraception includes one of the following:

- Systemic contraceptives (combined birth control pills, injectable/implant/insertable hormonal birth control products, transdermal patch)
- Intrauterine device (with or without hormones)
- Barrier methods of contraception (male condom with spermicide, female condom, cervical cap, diaphragm, contraceptive sponge)
- Male partner vasectomized at least 6 months prior to the first study product administration

Or

1. Is of childbearing potential and agrees to abide by true abstinence from heterosexual intercourse, when this is in line with the preferred and usual lifestyle (not periodic abstinence)

Or

1. Male partner has had a vasectomy less than 6 months prior to dosing, and agrees to use an additional acceptable contraceptive method from the first study product administration through to at least 30 days after the last dose of study product

Or

1. Is of non-childbearing potential, defined as surgically sterile (i.e., has undergone complete hysterectomy, bilateral oophorectomy, or tubal ligation) or is in a postmenopausal state (i.e., at least 1 year without menses without an alternative medical condition prior to the first study product administration)
2. Aged at least 19 years but not older than 55 years
3. Body mass index (BMI) within 18.5 kg/m^2^ to 30.0 kg/m^2^, inclusively
4. Minimal body weight of 50 kg
5. Primary tobacco product used in the last 6 months is combustible cigarettes or roll‑your‑own cigarettes
6. A smoker of 10 cigarettes or more >6 mg ISO tar per day who has smoked for at least 6 months prior to the first study product administration
7. Stated willingness to abstain from nicotine and tobacco products (except for the study products provided) from 24 hours prior to the first study product administration until the end of the study
8. Positive urine cotinine test (≥200 ng/mL) at screening and prior to the first study product administration
9. Successful completion of the training session for study product use prior to the first study product administration (subject is able to follow the instructions and does not experience significant adverse events during the training session)
10. Clinical laboratory values within the laboratory's stated normal range; if not within this range, they must be without clinical significance, as determined by an investigator
11. Have no clinically significant diseases captured in the medical history or evidence of clinically significant findings on the physical examination (including oral mucosa examination and vital signs) and/or ECG, as determined by an investigator

# **Exclusion Criteria**

1. Female who is lactating at screening
2. Female who is pregnant according to the pregnancy test at screening or prior to the first study product administration
3. Presence of any tongue piercings or history of any tongue piercings in the last 90 days prior to the first study product administration
4. Presence of braces, partials, dentures or any dental work that could, in the opinion of an investigator, affect the conduct of the study (including missing molars)
5. Presence or history of significant form of oral and/or pharyngeal inflammation, oral lesions and/or gum disease or temporomandibular joint dysfunction
6. History of significant hypersensitivity to any excipients of the formulations as well as severe hypersensitivity reactions (like angioedema) to any drugs
7. Presence or history of significant gastrointestinal, liver or kidney disease, or surgery that may affect drug bioavailability
8. History of significant cardiovascular, pulmonary, hematologic, neurological, psychiatric, endocrine, immunologic or dermatologic disease
9. Presence of clinically significant ECG abnormalities at the screening visit, as defined by medical judgment
10. Maintenance therapy with any drug (with the exception of hormonal contraceptives or hormone replacement therapy) or significant history of drug dependency or alcohol abuse (> 3 units of alcohol per day, intake of excessive alcohol, acute or chronic)
11. Any clinically significant illness in the 28 days prior to the first study product administration
12. Use of any prescription drugs (with the exception of hormonal contraceptives or hormone replacement therapy) in the 28 days prior to the first study product administration, that in the opinion of an investigator would put into question the status of the participant as healthy
13. Use of pseudoephedrine in the 7 days prior to the first study product administration
14. Use of any medication or substance that aids in smoking cessation, including but not limited to any nicotine replacement therapy (e.g., nicotine gum, lozenge, patch), varenicline (Chantix^®^), bupropion (Wellbutrin^®^, Zyban^®^), or Lobelia extract in the 28 days prior to the first study product administration
15. Any history of tuberculosis
16. Positive test result for alcohol and/or drugs of abuse at screening or prior to the first product administration
17. Positive screening results to HIV Ag/Ab Combo, Hepatitis B surface Antigen (HBsAG (B) (hepatitis B)) or Hepatitis C Virus (HCV (C)) tests
18. Inclusion in a previous group for this clinical study
19. Intake of an Investigational Product (IP) in any other clinical trial in the 28 days prior to the first study product administration
20. Donation of 50 mL or more of blood in the 28 days prior to the first study product administration
21. Donation of 500 mL or more of blood (Canadian Blood Services, Hema-Quebec, clinical studies, etc.) in the 56 days prior to the first study product administration
22. Postponement of a decision to quit using tobacco- or nicotine-containing products in order to participate in this study
23. Previously attempted to quit using tobacco- or nicotine-containing products in the 28 days prior to the first study product administration.

# **Lifestyle and/or Dietary Requirements**

- Subjects will be prohibited from consuming food or beverages containing grapefruit and/or pomelo for 7 days prior to the period 1 study product administration and during the study.
- Subjects will be prohibited from consuming alcohol for 48 hours prior to the period 1 study product administration and during the study.
- Subjects will be prohibited from consuming food or beverages containing xanthines (i.e., tea, coffee, cola drinks, energy drinks or chocolate) for 48 hours prior to the period 1 study product administration and during the study.
- Subjects will eat only the food provided by the study site during confinement at the clinical research unit (CRU).
- Subjects will be prohibited from consuming cannabinoids for 14 days prior to the period 1 study product administration and during the study.
- Subjects will abstain from nicotine and tobacco products (except for the study products provided) for 24 hours prior to the period 1 study product administration and until 12 hours after the last product administration of the study.
- Female subjects of childbearing potential will have to take appropriate measures to prevent pregnancy for at least 28 days prior to the first study product administration (in period 1), during the study and for at least 30 days after the last study product administration (period 3). It is the participant’s responsibility to notify the CRU if a pregnancy occurs from the end of their study participation until 30 days after the last study product dosing.
- Subjects will undergo a complete search of their personal belongings, including jackets/coats, if applicable, and personal belongings will be decontaminated at admission to the clinical site. Subjects’ personal belongings will be decontaminated again at 24 hours prior to product administration of period 1. Subjects will not have access to their personal belongings from 24 hours prior to the product administration of period 1 until the end of the confinement period (with the exception of decontaminated personal belongings that are mandatory to the subject).
- Subjects will be required to take a shower and to wash their hair at admission to the clinical site and at 24 hours prior to the product administration of period 1.
- Subjects will be required to wear clothing provided by the clinical site for the duration of the confinement period.

# **Schedule of activities**

Table 1. Schedule of Activities

|  | **Screening** |  | | **Period 1** | **Period 2** | **Period 3** | **End of Study**^1^ |
| --- | --- | --- | --- | --- | --- | --- | --- |
| **Day** | **-28 to -2** | **-2** | **-1** | **1** | **2** | **3** | **3** |
| Informed Consent^2^ | X |  |  |  |  |  |  |
| Eligibility Criteria Review | X | X |  |  |  |  |  |
| Demographics | X |  |  |  |  |  |  |
| Medical History | X |  |  |  |  |  |  |
| Smoking Habit Questioning | X |  |  |  |  |  |  |
| Admission^3^ |  | X |  |  |  |  |  |
| Vital Signs^4^ | X | X |  | X | X | X |  |
| Physical Examination^5^ | X | X |  |  |  |  | X |
| Examination of Oral Mucosa^6^ | X | X |  | X | X | X |  |
| Product Appreciation Questionnaire^7^ |  |  |  | X | X | X |  |
| Clinical Laboratory Tests^8^ | X |  |  |  |  |  | X |
| Serology | X |  |  |  |  |  |  |
| 12-lead ECG | X |  |  |  |  |  |  |
| Alcohol, Cotinine and Drugs of Abuse Screen | X | X |  |  |  |  |  |
| Serum Pregnancy Test (females only) | X | X |  |  |  |  | X |
| Randomization |  |  |  | X |  |  |  |
| Training Session^9^ |  | X | X |  |  |  |  |
| Study Product Administration^10^ |  |  |  | X | X | X |  |
| Blood sampling for PK^11^ |  |  |  | X | X | X |  |
| Discharge^12^ |  |  |  |  |  | X |  |
| AE Monitoring^13^ | X | X | X | X | X | X | X |
| Concomitant Medication Recording | X | X | X | X | X | X | X |

^1^ End of study procedures will be performed following completion of period 3, or as soon as possible after the last study treatment administration for subjects who are withdrawn.

^2^ The latest version of the consent form must be signed prior to subject's inclusion (prior to the first product administration).

^3^ Admission to the clinical site will occur 36 hours prior to the first product administration.

^4^ Vital signs at screening, at admission to the clinical site and prior to each product administration will include blood pressure, pulse rate and body temperature. Vital signs following each product administration will include blood pressure and pulse rate. On-study vital signs scheduled time points for each treatment period are: prior to product administration and at approximately 1 and 12 hours following each product administration.

^5^ Complete physical examination at screening. Symptom-oriented physical examination will be performed as needed, at admission to the clinical site and end of study.

^6^ Oral mucosa examination will be performed at screening and at admission to the clinical site. On-study oral mucosa examination scheduled time points for each treatment period are: prior to product administration, at the time of end of use (Test and Reference-1) or at the time of complete disintegration (Reference-2), and at 1 and 2 hours following each product administration.

^7^ Subjects will answer a short questionnaire on their appreciation of each product shortly following the end of use of each study product.

^8^ Clinical laboratory parameters are detailed in **Table 4**.

^9^ A training session using the Test, Reference-1 and Reference-2 will be conducted within 36 to 24 hours prior to the first product administration. There will be a minimum of 60 minutes between the use of each product.

^10^ The product administration start time of each period will be separated by at least 24 hours.

^11^ Blood sampling time points for PK determinations for each treatment period are specified in blood sampling for nicotine PK assessment section of the manuscript.

^12^ Discharge from the clinical site will occur after the 12-hour PK blood sample of Period 3.

^13^ AEs will be monitored until discharge from the clinical site on Day 3.

# **Safety Assessments**

Safety assessments will include symptom-oriented physical examination, oral mucosa examination, vital signs, clinical laboratory tests, and AE monitoring. Additional safety measurements may be performed at the discretion of an investigator for reasons related to subject safety.

The physician in charge will be present at the clinical site for at least the first 4 hours following each product administration and will remain available at all times throughout the study.

## Medical History

The medical history at screening will include all queries by the medical and clinical staff related to the subject’s well-being and history of relevant past medical events/experiences. Medical history will include all demographic data (age, gender, race, body weight, height, and BMI) and baseline characteristics. Alcohol and smoking habits will also be recorded.

## Physical Examination

A physical examination will be performed by a medically qualified and licensed individual as scheduled in **Table 1**.

The physical examination will include a general review of the following body systems (at minimum): head and neck, cardiovascular, respiratory, abdomen, brief neurological and general appearance, unless a symptom-oriented physical exam is indicated.

The physical examination performed at screening will also include a general mouth examination.

## Vital Signs

Vital signs will be measured as scheduled in **Table 1**. Vital signs at screening, at admission to the clinical site and prior to each product administration will include blood pressure, pulse rate and body temperature. Vital signs following each product administration will include blood pressure and pulse rate.

On-study time points for vital sign measurements are presented in **Table 3**.

Table 3. Vital Sign Recording Schedule

| **Vital Sign Recording - Scheduled Time Points** |
| --- |
| Prior to dosing |
| 1 and 12 hours postdose |

## 12-Lead Electrocardiogram

A twelve-lead ECG will be performed at screening as scheduled in **Table 1**.

## Laboratory Evaluations

Laboratory evaluations will be performed as scheduled in **Table 1**.

The laboratory evaluations to be conducted for this study are presented in **Table 4**. Additional clinical laboratory tests may be performed by the medical laboratory as part of larger standard test panels (not required for subject safety).

The physician in charge or delegate will assess each abnormal value to determine if it is clinically significant. Postdose clinically significant laboratory values will be reported as AEs, if applicable, as judged by the physician in charge or delegate. Verification of collection for all laboratory test panels will be collected in the clinical database.

Table 4. Clinical Laboratory Evaluations

| Clinical Laboratory Test Panel | Description |
| --- | --- |
| General biochemistry^1^: | Sodium, potassium, chloride, glucose, creatinine, bilirubin total, alkaline phosphatase, alanine aminotransferase and albumin |
| Hematology: | White cell count with differential (absolute values of neutrophil, lymphocyte, monocyte, eosinophil, and basophil), red cell count, hemoglobin, hematocrit, mean corpuscular volume, and platelet count |
| Serology^2^: | Human immunodeficiency virus (HIV) Ag/Ab Combo, Hepatitis B (HBsAg (B)) and Hepatitis C (HCV (C)) |
| Urinalysis^2^: | Color, clarity, specific gravity, pH, leukocyte, protein, glucose, ketones, bilirubin, blood, nitrite, urobilinogen. Microscopic examination will only be performed if the dipstick test is outside of the reference range for leukocyte, blood, nitrite or protein |
| Urine drug screen: | Amphetamines, barbiturates, cannabinoids, cocaine, cotinine, opiates and phencyclidine |
| Serum Pregnancy test: | To be performed for all female subjects |
| Alcohol screen: | Alcohol breathalyzer or urine screen |

^1^ At the End of Study, only alanine aminotransferase and creatinine will be measured.

^2^ Performed at the screening visit only.

## Oral Mucosa Examination

Examination of the oral mucosa (including local irritancy) will be performed at screening, at admission to the clinical site and during each study period as scheduled in **Table 1**. On-study time points for oral mucosa examination are presented in **Table 5**.

Table 5. Oral Mucosa Examination Schedule

| **Oral Mucosa Examination - Scheduled Time Points** |
| --- |
| Prior to dosing |
| At the time of end of use (Test* and Reference-1) or at the time of complete disintegration (Reference‑2) |
| 1 and 2 hours postdose |

*For the Test product, the end of use timepoint (expected to occur approximately 60 minutes following Test product administration) will correspond to the 1-hour postdose timepoint. In this case, the oral mucosa examination will only be performed once at 1 hour postdose and this will not be noted as a protocol deviation.

# **ADVERSE EVENTS**

## Definitions

An AE is defined as any untoward medical occurrence in a subject administered an IP and which does not necessarily have a causal relationship with the treatment. An AE can therefore be any unfavorable and unintended sign (including a clinically significant abnormal clinical laboratory finding, for example), symptom, or disease temporally associated with the use of an IP, whether or not related to the IP.

Adverse Drug Reactions are defined as: all noxious and unintended responses to an IP related to any dose. The phrase "responses to an IP" means a causal relationship between an IP and an AE is at least a reasonable possibility, i.e., the relationship cannot be ruled out.

An AE may be:

- A new illness,
- Worsening of a concomitant illness,
- An effect of the study drug including comparator(s); it could be an abnormal clinical laboratory value as well as a significant shift from baseline within normal range which an investigator considers to be clinically important.

Surgical procedures themselves are not AEs. They are therapeutic measures for conditions that required surgery. The condition for which the surgery is required is an AE, if it occurs or is detected during the study period. Planned surgical measures permitted by the clinical study protocol and the conditions(s) leading to these measures are not AEs, if the condition(s) was (were) known before the start of study treatment. In the latter case, the condition should be reported as medical history.

A serious adverse event (SAE) or reaction is any untoward medical occurrence that at any dose:

- Results in death,
- Is life-threatening,
- Requires inpatient hospitalization or prolongation of existing hospitalization,
- Results in persistent or significant disability or incapacity (defined as a substantial disruption of a person’s ability to conduct normal life functions),
- Is a congenital anomaly or birth defect,
- Is an important medical event (including development of drug dependence or drug abuse) that may jeopardize the subject or may require intervention to prevent one of the other outcomes listed above (according to medical judgment of an investigator)

## Severity Assessment

All AEs will be graded as mild, moderate, or severe according to the following definitions:

Mild: Causing no limitation of usual activities; the subject may experience transient slight discomfort

Moderate: Causing some limitation of usual activities; the subject may experience annoying discomfort

Severe: Causing inability to carry out usual activities; the subject may experience intolerable discomfort or pain

Every effort will be made to obtain an adequate evaluation of the severity.

## Causality Assessment

An investigator will determine the relationship of any AE to the study product using the guidelines presented in **Table 6**.

Table 6. Adverse Event Relationship to Study Product

| Relationship to Product | Comment |
| --- | --- |
| Reasonable Possibility | A temporal relationship exists between the adverse event (AE) onset and administration of the investigational product that cannot be readily explained by the subject’s clinical state or concomitant therapies.  Furthermore, the AE appears with some degree of certainty to be related, based on the known therapeutic and pharmacologic actions or AE profile of the investigational product.  In case of cessation or reduction of the dose the AE may abate or resolve and it may reappear upon rechallenge. |
| No Reasonable Possibility | Evidence exists that the AE has an etiology other than the investigational product.  For serious AEs, an alternative causality must be provided (e.g., preexisting condition, underlying disease, intercurrent illness, or concomitant medication). |
